# Repeated Exposure of Nanostructured Titanium to Osteoblasts with Respect to Peri-Implantitis

**DOI:** 10.3390/ma13030697

**Published:** 2020-02-04

**Authors:** Vaclav Babuska, Jana Kolaja Dobra, Ludek Dluhos, Jana Dvorakova, Jana Moztarzadeh, Daniel Hrusak, Vlastimil Kulda

**Affiliations:** 1 Department of Medical Chemistry and Biochemistry, Faculty of Medicine in Pilsen, Charles University, Karlovarska 48, 301 66 Pilsen, Czech Republic; jana.dobra@lfp.cuni.cz (J.K.D.); jana.dvorakova@lfp.cuni.cz (J.D.); jana.moztarzadeh@seznam.cz (J.M.); vlastimil.kulda@lfp.cuni.cz (V.K.); 2 Timplant s.r.o., Sjednoceni 77/1, 72525 Ostrava, Czech Republic; timplant@timplant.cz; 3 Department of Stomatology, Faculty of Medicine in Pilsen, University Hospital and Charles University, alej Svobody 80, 301 00 Pilsen, Czech Republic; hrusak@fnplzen.cz

**Keywords:** nanostructured titanium, peri-implantitis, osteoblasts proliferation, biocompatibility

## Abstract

Titanium offers excellent biocompatibility and extraordinary mechanical properties. As a result, it is used as a material for dental implants. Implants infected by peri-implantitis can be cleaned for successful re-osseointegration. Optimal surface properties, such as roughness and wettability, have a significant impact on cell adhesion. The aim of this study was to evaluate the adhesion and proliferation of osteoblasts on the surface of repeatedly cleaned nanostructured titanium samples. Human osteoblast-like cells MG-63 were seeded on nanostructured titanium specimens manufactured from rods produced by the equal channel angular pressing. For surface characterization, roughness and wettability were measured. Cell adhesion after 2 h as well as cell proliferation after 48 h from plating was assessed. We have found that this repeated cleaning of titanium surface reduced cell adhesion as well as proliferation. These events depend on interplay of surface properties, such as wettability, roughness and topography. It is difficult to distinguish which factors are responsible for these events and further investigations will be required. However, even after the several rounds of repeated cleaning, there was a certain rate of adhesion and proliferation recorded. Therefore the attempts to save failing implants by using in situ cleaning are promising.

## 1. Introduction

Titanium is a commonly used implant material in the field of biomedical applications. Its tremendous potential is based on its low density, high level of corrosion resistance with good plasticity, resistance to body fluids, non-magneticity and non-toxicity. Spontaneous formation of titanium dioxide film over its surface results in excellent biocompatibility [1,2,3]. A never-ending pursuit of more successful materials has led to new methods for improving different properties of the metals used in bioimplantology. One way to improve the mechanical properties of commercially pure titanium (cpTi) is to refine its structure to form ultra-fine grained (UFG) titanium. Refinement of microstructure is associated with a decrease of grain size to hundreds of nanometers, resulting in an improvement of strength levels. Among the methods for considerable grain refinement, severe plastic deformation (SPD) is the most popular. One of the methods leading to bulk and fully-dense nanostructured titanium (nTi) is equal channel angular (ECA) pressing [4]. The goal of the ECA pressing method is to introduce a simple shear strain when the rod of titanium material passes through the plane between two channels under a different angle. The material exiting the channel has the same cross-section and thus can be passed through the channel more times. Repetitive pressing allows rotation of the billet between the passes and leads to different microstructures [5].

Peri-implantitis is defined as an inflammatory process affecting tissues around an osseointegrated implant accompanied by progressive loss of supporting bone. It negatively affects the prognosis of the implant survival. The loss of surrounding bone changes the ratio between extra- and intra-alveolar parts of the implant, thereby reducing the amount of chewing pressure transferred from the implant to the bone during physiological load [6]. Although peri-implantitis has been described as a polymicrobial infection associated with pathogenic bacterial strains including *Porphyromonas gingivalis*, *Tannerella forsythia*, *Fusobacterium nucleatum*, and *Treponema socranskii* [7,8], the growth of these late colonizers is primarily dependent on biofilm formation on the implant surface by early-colonizing strains. The pioneer oral bacterial species are mainly streptococci, like *Streptococcus salivarius*, *Streptococcus mitis*, and *Streptococcus oralis*. Oral streptococci produce an arsenal of adhesive molecules that allow them to efficiently colonize different surfaces in the mouth and prepare a suitable environment for pathogenic bacteria [9].

Currently, no method for the treatment of implants affected by peri-implantitis has been standardized. The basic principle of treatment is based on either removal of or trying to save failing implants depending on the area of affected surface [10,11]. The difference of re-osseointegration potential between contaminated and new implants was described by Levin et al. [12]. Failed implants were retrieved from the infected bone sites and such contaminated implants were inserted into new sockets. New implants were inserted into peri-implantitis sockets. It was found that osseointegration was achieved both in the infected sites with new implants and around contaminated implants in the new sockets. In contrast, Persson et al. [13] reported failure of the significant re-osseointegration for implant surfaces exposed to bacterial contamination. These results could suggest the importance of the findings, if the restoring of the implant surfaces to their original condition could determine successful re-osseointegration [14]. Plenty of articles discuss cleaning of the biofilms on the initially microbially exposed surfaces of titanium implants [15,16] or the effect of supportive care in the prevention of peri-implant diseases [17], but until now there has been limited information concerning cellular behavior on used and consecutively cleaned surfaces.

The aim of this study was to evaluate repeated adhesion and proliferation of osteoblasts on the surface of nanostructured titanium samples after cleaning procedures with the intention of determining the suitability of such nanomaterials for repeated use in dental implantology.

## 2. Materials and Methods

### 2.1. Material

All samples (flat titanium discs, 3.0 mm in height and 5.5 mm in diameter) were processed by turning from grade 4 nTi rods, which were produced by using the ECA pressing method on a continuous extrusion machine (Conform 315i, BWE Ltd., Ashford, UK). For further ECA processing details, see the work published by reference [18]. The flat surface was then treated by polishing with abrasive paper to produce smooth surfaces with roughness Ra < 0.1 µm. Samples were then acid etched to receive roughness Ra in the range between 0.25 and 0.55 µm. The procedure of acid etching has been described elsewhere [19].

All twenty samples were then divided in four groups of about five pieces with non-significant differences in roughness (Table 1). Each implant was sterilized by rinsing in 70% ethanol before first usage. Finally the implants were rinsed in deionized water and left to dry.

**Figure 1:**
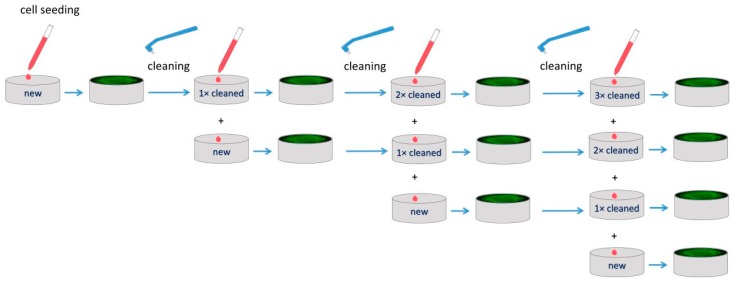
Scheme of the experiment: cell seeding on new as well as repeatedly cleaned titanium samples.

Each implant was cleaned and sterilized before the repeated usage—the scheme of the experiment is shown in Figure 1. The procedure contained incubation in a trypsin solution (0.25% (w/v) Trypsin with 0.53 nM EDTA) (PAA Laboratories GmbH, Pasching, Austria) (37 °C, 30 min) followed by thorough cleaning with toothbrush TePe Compact Tuft (TePe, Malmö, Sweden) and rinsing in both 70% ethanol and deionized water. Finally the samples were left to dry.

### 2.2. Characterization of Surfaces

A mechanical contact profilometer Surtronic 25 (Taylor Hobson, Leicester, UK) was used for measurement of surface roughness of each sample. Each sample was analyzed four times. Between each measurement, the sample was rotated about 45° along an axis passing through the center of the cylinder. The surface roughness was then expressed as the arithmetical mean roughness Ra and root mean square Rq. The values were captured with a diamond-tipped stylus (radius 5 µm) at a traverse speed of 1 mm/s. An average value was expressed as the mean surface roughness for each specimen.

Estimation of surface wettability was performed by using the sessile drop contact angle method (LeicaS9i, Leica Microsystems, Wetzlar, Germany). The contact angle was measured 3 s after placing a 1 µL droplet of ultrapure water on the surface. An average from three measurements was recorded as the mean value.

### 2.3. Cell Cultures

Human osteoblast-like cell line MG-63 (ECACC 86051601) (Sigma Aldrich, St. Louis, MO, USA), obtained from an osteosarcoma of a 14-year-old boy, was cultivated in Dulbecco’s Modified Eagle’s Medium (DMEM, Biosera Europe, Nuaille, France) supplemented with 10% (v/v) fetal bovine serum (FBS, Biosera Europe, Nuaille, France), 100 U/mL penicillin and 100 mg/mL streptomycin (PAA Laboratories GmbH, Pasching, Austria) as well as 2.5 mM stable glutamine (Diagnovum GmbH, Ebsdorfergrund, Germany) at 37 °C under 5% CO_2_ in a humidified incubator. Culture media were refreshed as needed.

### 2.4. Cell Adhesion and Proliferation

Cell adhesion after 2 h as well as cell proliferation after 48 h from seeding was evaluated by using CCK-8 (Cell Counting Kit-8, Bimake, Munich, Germany) as stated in the manufacturer’s instructions with volume modifications. The working procedure with cells was similar to that used previously [2], with some modifications. Instead of 96-well plates, we used 48-well plates. The seeding density was 250,000 cells/mL. After 2 and 48 h of incubation, the CCK-8 test was applied. 

After 2 h from plating, cells were rinsed with phosphate-buffered saline (PBS) and incubated with 550 μL CCK-8 working solution (50 μL CCK-8 solution + 500 μL culture media) at 37 °C. After 16 hours, 110 μL aliquots of working solution were transferred to a 96-well plate and the absorbance at 450 nm was evaluated using a microplate reader Synergy H1 (Biotek, Winooski, VT, USA). Simultaneously, the culture media were changed in each well and the incubation was continued. After 48 h from plating, 50 µL of CCK-8 solution was added to each well of the 48-well plate and the wells were incubated for 16 h at 37 °C. The microplate reader Synergy H1 (Biotek, Winooski, VT, USA) was used for measuring the absorbance at 450 nm. Cell adhesion as well as proliferation was expressed as a percentage of the positive control. 

### 2.5. Cell Staining

Cultured cells were stained with CellTracker™ Green (Molecular Probes Inc., Eugene, OR, USA) and NucBlue® Live ReadyProbes® Reagent (Thermo Fisher Scientific, Waltham, MS, USA) according to the manufacturer’s instructions. Briefly, 2 drops of NucBlue® Live ReadyProbes® Reagent per milliliter of media were added and the cells were incubated for another 30 min at 37 °C. Finally, the medium was changed with Live Cell Imaging Solution (Thermo Fisher Scientific, Waltham, MS, USA). The cells were observed by using an Olympus CKX41 inverted fluorescent microscope (Olympus, Hamburg, Germany) at 400× magnification.

Cells were also stained with crystal violet. The cells were washed with PBS and fixed with 2.5% glutaraldehyde in PBS (pH = 6.7–7.1) for 30 min at room temperature. After fixation, cells were washed with PBS again and stained with 0.5% solution of crystal violet for 20 min at room temperature. Then, staining solution was removed and cells were rinsed with deionized water three times. Cells were observed and photographed after drying under microscope Leica S9i (Leica Microsystems, Wetzlar, Germany).

### 2.6. Statistical Analysis

All experiments were performed using five samples per group. Results are presented as mean ± SD. Four independent experiments with quadruplicate measurement of absorbance were performed. Cell adhesion and proliferation was analyzed by ANOVA (analysis of variance) or a two-tailed unpaired t-test where appropriate. If ANOVA denoted a significant difference (P < 0.05) further statistical comparisons were performed by post hoc Tukey test with the value of significance P < 0.05. The SigmaPlot 12.5 software (Systat Software Inc., San Jose, CA, USA) was used for all statistical analysis.

## 3. Results

### 3.1. Roughness and Wettability

The surface roughness quantified by the arithmetical mean roughness Ra and root mean square Rq is shown in Table 1 for each group of samples. The Ra and Rq values for acid etched surfaces of new implants were between 0.252–0.542 µm and 0.333–0.816 µm, respectively. No significant differences were found between the groups of nanostructured materials. Also the cleaning had no significant impact on the surface roughness. The Ra and Rq values for surfaces of cleaned implants were between 0.255–0.397 µm and 0.345–0.576 µm, respectively.

The mean wettability of all groups of new implants is shown in Table 1. The wettability of group C was significantly lower than the wettability of groups A and D with p = 0.0207 and p = 0.0061, respectively. 

The cleaning increased the wettability of the implant surface significantly compared to new implants. After the first cleaning, the wettability increased significantly (p = 0.0433). The second cleaning cycle also significantly increased the wettability (p = 0.0035). The third and fourth cleaning cycles did not have a significant additional effect on wettability.

### 3.2. Cell Adhesion and Proliferation

**Figure 2:**
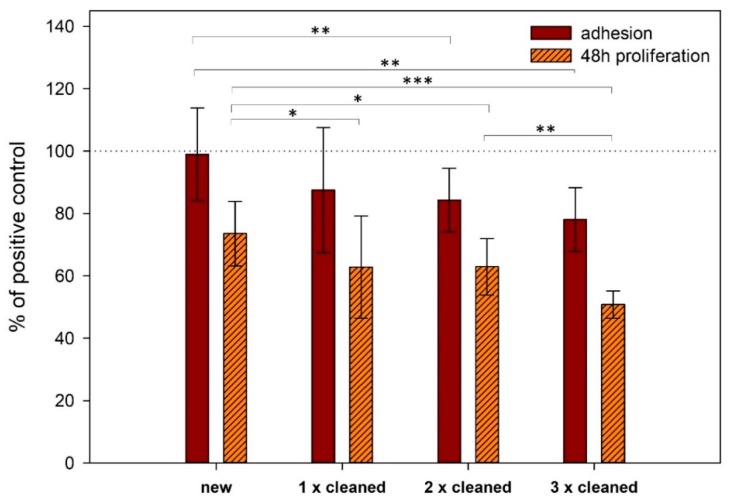
Comparison of cell adhesion and proliferation on the surface of new and different times cleaned titanium samples assessed by CCK-8 assay. The standard errors were calculated from all measurements (new—20 samples, 1× cleaned—15 samples, 2× cleaned—10 samples and 3× cleaned—5 samples). Data expressed as a percentage of positive control. Error bars indicate means ± standard deviations. Asterisks represent statistical significance between respective groups (* p < 0.05, ** p < 0.01, *** p < 0.001).

**Figure 3:**
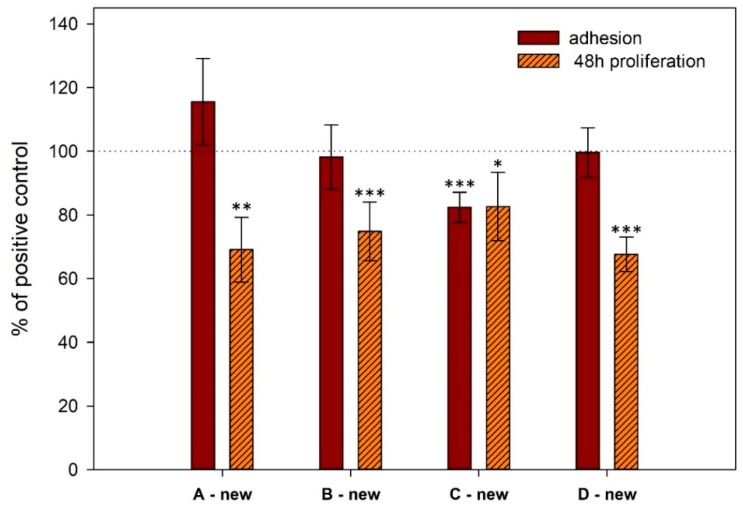
Comparison of cell adhesion and proliferation on the surface of new titanium samples assessed by CCK-8 assay in four independent experiments. The standard errors were calculated from five replicates. Data expressed as a percentage of positive control. Error bars indicate means ± standard deviations. Asterisks above the bars indicate significant differences (* p < 0.05, ** p < 0.01, *** p < 0.001) between titanium samples and positive controls.

The cell adhesion on titanium surface did not differ significantly between new implants and positive control (cultivation plastic) (Figure 2 and Figure 3). There was no significant difference between three of the four experiments performed; in the fourth experiment (sample Cnew), significantly lower cell adhesion compared with control was observed, as shown in Figure 3.

**Figure 4:**
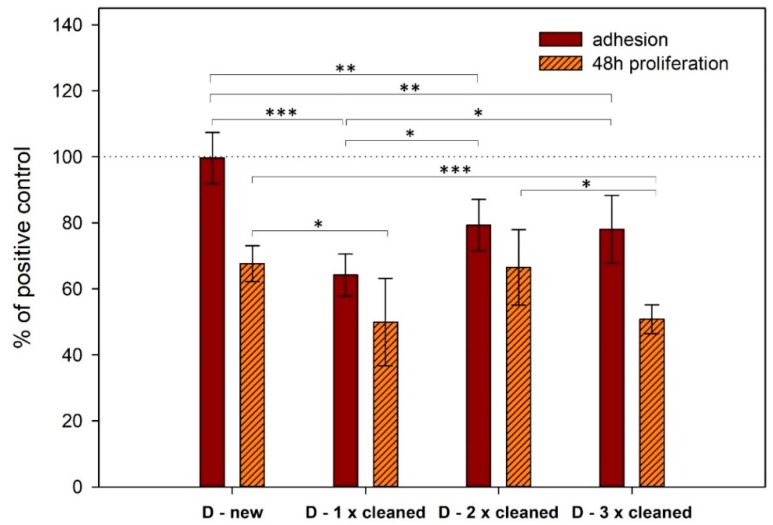
Comparison of cell adhesion and proliferation on the surface of new and different times cleaned same titanium samples assessed by CCK-8 assay. The standard errors were calculated from five replicates. Data expressed as a percentage of positive control. Error bars indicate means ± standard deviations. Asterisks represent statistical significance between respective groups (* p < 0.05, ** p < 0.01, *** p < 0.001).

When the cleaned implants were used, adhesion slightly decreased upon repetitive cleaning (Figure 2). When we compared cell adhesion on the surface of new titanium samples and the same ones which were cleaned using a different method, we saw different outcomes (Figure 4). The biggest difference was between the new and only once cleaned surface. We found that the difference narrowed with additional cycles of cleaning.

On new implants, proliferation is significantly lower than on cultivation plastic (control) (Figure 3). There is no significant difference between three of the four experiments performed; in the fourth experiment (sample Cnew), significantly higher proliferation was observed compared to the last experiment (sample Dnew) (p = 0.023).

The further decrease in the proliferation rate can be seen when cleaned implants were used (Figure 2). A significant decrease in this rate can be seen between new implants and ones that were cleaned once (p = 0.024), between new implants and ones that were cleaned twice (p = 0.010) and between new implants and ones that were cleaned three times (p < 0.001) (Figure 2). 

When we compared cell proliferation on the surface of new implants and older ones that were cleaned a different number of times, we saw a small discrepancy in ones that were cleaned two times (Figure 4).

### 3.3. Cell Morphology

**Figure 5:**
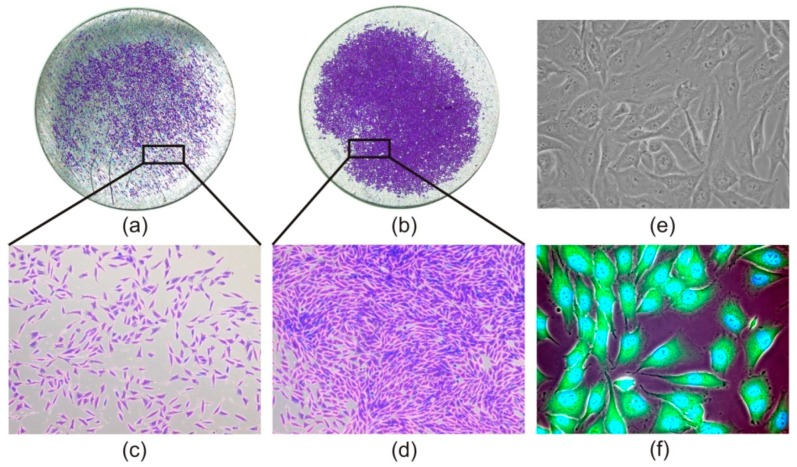
Microscopic images of cell morphology. Human osteoblast like cells MG-63 stained with crystal violet on nanostructured titanium sample in magnification 20× (**a**,**b**) and 100× (**c**,**d**). Native osteoblasts in bright field on tissue culture plate (**e**) and fluorescence image of MG-63 cells on tested titanium material (**f**), both in 400× magnification.

In order to compare morphology of the MG-63 cells grown on the nanostructured titanium material and on cultivation plastic, we stained the cells with CellTracker™ Green and NucBlue® Live ReadyProbes® Reagent as well as with crystal violet. The staining of the cells grown on titanium samples was necessary because unstained cells were not visible. Microscopic observation revealed that as early as after 2 h, osteoblasts presented normal MG-63 morphology and an adherent spindle shape of fibroblast phenotype (Figure 5a,c). The morphology of the cells did not change during the next 48 h (Figure 5b,d).

## 4. Discussion

Peri-implantitis is one of the most frequent complications of the implant osseointegration. The standard management of patients includes either preservation or removal of failing implants [20]. In the case of removal, the wound heals for months before a new implant can be used. Nowadays, efforts are being made to achieve successful re-osseointegration of failing implants affected by bacterial contamination by cleaning in situ in the oral cavity without conducting implant removal. This helps to reduce postoperative complications while providing an increased lifespan for biomedical implants [21].

In this study, we focused on an in vitro evaluation of how the standard repeated cleaning procedure influenced the adhesion and proliferation of osteoblasts on the surface of nanostructured titanium samples. We have found that this repeated cleaning reduces cell adhesion as well as cell proliferation on titanium surface. Optimal surface properties, such as wettability, topography and chemistry have significant roles in osteoblastic cell adhesion [22], which is critical for the clinical success of dental implants. It is, however, difficult to distinguish which of these factors is responsible for the specific behavior of cells, as surface properties are interdependent on each other [23].

Tested grade 4 nTi samples were divided into groups with similar surface roughness. The only significant difference in surface characteristics, which we determined, was the lower wettability of one of the studied groups. In this group, we saw worse osteoblast cells adhesion to the surface. However, we recorded that increase in wettability was associated with worse cell adhesion on the surfaces of repeatedly cleaned samples. This demonstrates that the influence of wettability on cell adhesion is not straightforward. It has been shown by numerous studies that wettability is an important factor for cell adhesion. Shi et al. observed that the surface with a contact angle of around 40° can improve fibroblast adhesion [24]. Zhao et al. found that wettability and surface energy were key parameters in the adhesion and spreading of osteoblastic cells [25]. It is worth mentioning that in clinically used implants, the contact angles range widely [26] and the surface wettability has not been a focus of surface characterization studies yet [27,28]. 

For decades, physical chemistry has studied how wettability can be affected by adjustments of surface roughness [29,30,31,32]. Additionally to surface roughness, wettability caused by aqueous solutions is closely dependent on hydrophilicity of the surface. Some studies showed that hydrophilic surfaces support cell attachment and enhance cell proliferation, spreading and differentiation compared with hydrophobic surfaces [33,34,35]. Kim et al. observed a significant increase in the cell adhesion and proliferation on the titanium surface when the grain size was reduced to the submicrometer range, resulting in higher surface wettability and hydrophilicity [36].

Nevertheless, the role of the surface hydrophilicity of the material in determining the adhesion is still not fully understood. Le Guehennec et al. compared cell viability on titanium and plastic with similar hydrophilicity and the surface roughness. Their finding of lower cell viability on titanium surfaces may be explained by a different chemical composition [37]. The interaction of cells with artificial surfaces of different wettabilities began to be studied many decades ago. In 1985, van Wachem et al. reported the best adherence of human endothelial cells onto moderately wettable polymers [38]. 

It is known that cell adhesion to titanium materials is mediated by proteins adsorbed on the surface from the culture media or produced by the cells. Proteins of the extracellular matrix, such as fibronectin, vitronectin, collagen and laminin contain specific amino acid sequences that bind to cell-surface integrin receptors and so influence cell adhesion. Recently, it has seemed that moderately hydrophilic surfaces are optimal for the cell adhesion. Both extremes, in the form of hydrophobic and highly hydrophilic surfaces, interfere with adsorption of proteins mediating adhesion [39]. Also Webb et al. concluded that moderately hydrophilic surfaces promoted the highest levels of cell attachment [40]. Implants with optimal hydrophilicity have been proven to improve the initial blood contact to support osseointegration [41,42].

We assumed that change of wettability caused by repeated cleaning can be triggered by the adsorption of various molecules produced by the cells. The change of wettability itself is probably less important than the question of which molecules modified the surface. Some of them can promote cell adhesion while others impair it. A surface condition after repeated cleaning where roughness is maintained, wettability is improved and proteins produced by the bone cells of the original tissue persist on the implant surface could be beneficial for re-osseointegration.

## 5. Conclusions

We observed that repeated cleaning of nanostructured titanium surface reduced osteoblasts adhesion as well as proliferation. It is difficult to distinguish which factors are responsible for these events, and further investigations will be required. However, even after several times of repeated cleaning, there was a certain rate of adhesion and proliferation recorded. Therefore the attempts to save failing implant by carrying out in situ cleaning are promising.

## Figures and Tables

**Table 1:** Arithmetical mean roughness Ra and root mean square Rq values for all groups of new as well as cleaned samples. Wettability is expressed in the form of the angle between the tangent of the drop and the horizontal baseline of the solid surface.

Implants	Group A		Group B		Group C		Group D	
	New	1× Cleaned	New	2× Cleaned	New	3× Cleaned	New	4× Cleaned
**Ra (µm)**	0.358	0.316	0.359	0.322	0.393	0.342	0.400	0.346
**Rq (µm)**	0.504	0.462	0.506	0.443	0.565	0.473	0.521	0.517
**(°)**	75.9	71.3	77.9	63.6	81.1	68.8	72.5	65.6
